# Nottingham Eczema Severity Scoring tool can identify children at high risk of food allergy to cow’s milk, egg and peanut

**DOI:** 10.1186/2045-7022-5-S3-P127

**Published:** 2015-03-30

**Authors:** Lizalet Oosthuizen, M A Mc Aleer, R M Watson, G M O’Regan, A Byrne, G Crispino-O’Connell, A D Irvine

**Affiliations:** 1 Department of Clinical Nutrition and Dietetics, Our Lady’s Children’s Hospital, Dublin, Ireland; 2 Department of Dermatology, Our Lady’s Children’s Hospital, Dublin, Ireland; 3 Department of Immunology, Our Lady’s Children’s Hospital, Dublin, Ireland; 4 Children’s Research Centre, Our Lady’s Children’s Hospital, Dublin, Ireland

## Background

Atopic dermatitis (AD) and food allergy (FA) often co-exist in early childhood. Increased severity of AD and younger age of patients’ correlates directly with the presence of IgE mediated FA. There is an increased need for early identification of the high risk patient with AD and FA.

## Objectives

To determine if the Nottingham Eczema Severity Score (NESS) is a useful eczema scoring tool in the clinical setting to identify AD patients at high risk of FA in a large, single centre cohort attending our tertiary AD clinic.

## Methods

1354 patients were included. Eczema severity was determined using the validated NESS tool. All patients had specific IgEs performed on the day of assessment for cow’s milk, egg and peanut. Previously reported positive predictive decision points were used for the diagnosis of high risk IgE food hypersensitivity (HR IgE FHS) by CAP-FEIA measurements.

## Results

When HR IgE FHS were categorized by eczema severity, those with severe AD were more likely to have HR IgE to cow’s milk (x2 11.3, P < 0.001), egg (x2 6.5, P < 0.05) and peanut (x2 25.4, P < 0.0001) compared to those with mild to moderate AD. Those ≤ 2 years with severe AD were more likely to have multiple HR IgE FHS compared to those with mild to moderate AD for all combinations: cow’s milk and egg (x2 18.62, P < 0.0001); peanut and egg: (x2 24.72, P < 0.0001); cow’s milk and peanut (x2 16.37, P < 0.0001); cow’s milk, egg and peanut (x2 20.01, P < 0.0001).

## Conclusions

Children with severe AD referred to our dermatology department are likely to have HR IgE FHS to cow’s milk, egg and peanut and a high level of suspicion for these foods is needed upon evaluation. The NESS eczema scoring tool is useful to identify patients at high risk for FA.

